# National survey of pre-treatment HIV drug resistance in Cuban patients

**DOI:** 10.1371/journal.pone.0221879

**Published:** 2019-09-03

**Authors:** Liuber Y. Machado, Madeline Blanco, Laura S. López, Héctor M. Díaz, Marta Dubed, Neisy Valdés, Enrique Noa, Liodelvio Martínez, María T. Pérez, Dania M. Romay, Caridad B. Rivero, José Joanes, Isis Cancio, María I. Lantero, Mireida Rodríguez

**Affiliations:** 1 AIDS Research Laboratory, Mayabeque, Cuba; 2 Hermanos Ameijeiras Hospital, Havana, Cuba; 3 Department of STI/HIV/AIDS, Ministry of Public Health, Havana, Cuba; University of Cincinnati College of Medicine, UNITED STATES

## Abstract

**Background:**

The World Health Organization (WHO) recommends a method to estimate nationally representative pretreatment HIV drug resistance (PDR) in order to evaluate the effectiveness of first -line treatments. The objective of the present study was to determine the prevalence of PDR in Cuban adults infected with HIV-1.

**Materials and methods:**

A cross-sectional study in Cuban adults infected with HIV-1 over 18 years was conducted. The probability proportional to size method for the selection of municipalities and patients without a prior history of antiretroviral treatment during the period from January 2017 to June 2017 was used. The plasma from 141 patients from 15 municipalities for the determination of viral subtype and HIV drug resistance was collected. Some clinical and epidemiological variables were evaluated.

**Results:**

80. 9% of the patients corresponded to the male sex and 76.3% were men who have sex with other men (MSM). The median CD4 count was 371 cells / mm^3^ and the median viral load was 68000 copies / mL. The predominant genetic variants were subtype B (26.9%), CRF19_cpx (24.1%), CRF 20, 23, 24_BG (23.4%) and CRF18_cpx (12%). Overall, the prevalence of PDR was 29.8% (95%, CI 22.3–38.1). The prevalence was 12.8% (95%, CI 6.07–16.9) for any nucleoside reverse transcriptase inhibitor (NRTI), 23.4% (95%, CI 16.7–31.3) for any non-reverse transcriptase inhibitor (NNRTI) and 1.4% (95%, CI 0.17–5.03) for any protease inhibitor (PI). The most frequent mutations detected were K103N (12.9%), G190A (6.4%) and Y181C (4.8%).

**Conclusions:**

The NNRTI prevalence above 10% in our study indicates that the first-line antiretroviral therapy in Cuba may be less effective and supports the need to look for new treatment options that contribute to therapeutic success and help the country achieve the global goals 90-90-90 set forth by UNAIDS.

## Introduction

Highly active antiretroviral therapy (HAART) is one of the most significant advances in the control of the HIV / AIDS epidemic, because it has reduced morbidity and mortality in people living with HIV (PLHIV). However, because HAART is not capable of eradicating the virus from the organism, treatment must be for life [1]. The emergence and transmission of HIV variants resistant to antiretroviral drugs can have a negative impact on the success of short- or long-term treatment [2] and compliance with the third 90 of the goals 90-90-90 stated by UNAIDS for the year 2020, which is to achieve values of viral load not detectable in 90% of patients who are receiving HAART [3]. The WHO recommends that countries that have established antiretroviral therapy programs should establish surveillance systems for HIV resistance to antiretroviral (HIVDR) in PLHIV that receive HAART and those that are not treated, in conjunction with the monitoring of the early warning indicators of HIVDR. The purpose of the WHO strategy with HIVDR surveillance is to minimize the emergence of drug resistance, prolong the effectiveness of first and second line therapies, and improve the quality of life of PLHIV [4].

The HAART began in Cuba in 2001 with the employment in the first and second line of generic antiretroviral produced by the national biopharmaceutical industry, which has benefited to 83% of PLHIV (n = 18744) until December 2017 (MINSAP Computerized Registry). Several working groups have studied the situation of HIVDR in Cuba in untreated PLHIV at different times, which reported values of prevalence of HIVDR from 4% to 20.2% [5–9]. However, these investigations were not carried out using the sampling methodology described by the WHO to conduct national surveillance studies representative of HIVDR, which constitutes a methodological limitation according to the recommendations issued. National studies of HIVDR surveillance in pre-treatment PLHIV would allow health authorities to evaluate the effectiveness of the therapeutic regimens used, as well as the analysis of the effectiveness of interventions to face resistance.

In 2017, AIDS Research Laboratory and the National STI / HIV / AIDS Program of the Ministry of Public Health, with WHO advice, conducted a nationwide representative survey of Cuban patients with HIV-1 with the objective of estimating the prevalence of HIVDR in people who would start HAART.

## Materials and methods

### Survey design and sample collection

For the design of the national survey all patients diagnosed with HIV-1 infection, older than 18 years of age, and above who were initiating HAART and were antiretroviral therapy naive during the months January and June 2017, from the 168 municipalities of the country, were included. For sampling, the number of patients previously exposed to antiretroviral treatment and subsequent interruption for more than three months was unknown, so they were not included in the study design. Women with previous pregnancies were excluded from sampling.

The 10% of the municipalities with the lowest number of patients that would start HAART were eliminated, which guaranteed a sampling frame that represented 90%. The "Probability Proportional to Proxy Size (PPPS)" sampling was used to select 165 patients from 15 municipalities (S1 Table, S1 Fig). The informed consent of each patient who participated in the survey was obtained, prior to the sampling and collection of clinical and demographic data. 10 mL of whole blood was obtained in EDTA vacutainers and sent to AIDS Research Laboratory for the determination of plasma viral load and HIVDR.

### Ethics statement

The national survey was reviewed and approved by the Council of Researchs of AIDS Research Laboratory (CC-LISIDA-16-018). Ethical procedures were carried out in accordance with the requirements or standards of the Ministry of Public Health of the Republic of Cuba and the Ministry of Science, Technology and Environment which comply with the principles laid down in the Declaration of Helsinki for medical research in Human beings. This study was conducted with ethical clearance by the research on Human Subjects (Medical) Committee at the AIDS Research Laboratory, Cuba (CIE-LISIDA-16-04). Prior to sample collection, informed consent was obtained from each patient who participated in the study.

### HIV sequencing and drug resistance assessment

HIV RNA was extracted from 1 mL of plasma (QIAamp Viral RNA kit, QIAGEN, Valencia, CA, USA). The protease (PR) and reverse transcriptase (RT) regions were amplified by an “in house” method [10]. Sequences were obtained by chemistry of the GenomeLab^TM^ DTCS Quickstar kit (Beckman Coulter, USA) on a CEQ TM 8800 genetic analyzer (BeckmanCoulter, USA) and assembled using the Sequencher version 5.0 (GeneCodes, Inc., Ann Arbor, Michigan, USA). The subtype of HIV-1 was determined by REGA HIV-1 subtypingtool v 3.0 (http://dbpartners.stanford.edu:8080/RegaSubtyping/stanford-hiv/typingtool/) and was confirmed by phylogenetic analysis.

Sequences were aligned using ClustalW in Mega v7. Reference sequences for different HIV subtypes were obtained at Los Alamos HIV Sequence Database (www.hiv.lanl.gov) and included in the alignments. For the determination of possible recombinants and breakpoints, bootscaning was performed and the points of similarity between sequences were determined using the Simplot v 3.5 and RDP4 programs. Additionally, phylogenetic tree was constructed using the neighbor joining method and the genetic distance was estimated according to the Kimura two parameters. Bootstrap values were calculated based on 1000 replicates. The tree was visualized using the program FigTree v 1.1.2 (http://tree.bio.ed.ac.uk/software/figtree/).

Nucleotide sequences were analyzed using Stanford HIV database algorithm, available on the Stanford HIV database website (http://hivdb.stanford.edu/hivdb/by-mutations/). Any HIVDR was defined in sequences classified as low-, intermediate- or high-level resistance according to the Stanford HIVdb algorithm with respect to one or more antiretrovirals (ARVs). Drug resistance level was classified according to the Stanford Penalty Score as high (≥60), intermediate (30–59), or low (15–29). The surveillance mutations were defined per the 2009 WHO Surveillance Drug Resistance Mutations [11]. The sequences with mutations associated with resistance to ARVs were subjected to a phylogenetic analysis by constructing a tree using the maximum likelihood method (ML) and the GTR+G+I model. Possible clusters of transmission were identified using Cluster Picker, with a 1.5% genetic distance threshold [12].

To evaluate the quality control of the sequences, the WHO HIVDR tool (http://pssm.cfenet.ubc.ca/who_qc) was used.

### Statistical analysis

Statistical analyses were performed in GraphPad Prism 6 (San Diego, CA, USA). Comparisons between groups were performed using Fisher’s exact or χ^2^ test for categorical variables. For all prevalence calculations, 95% confidence intervals were calculated using the modified Wald method (p<0.05).

## Results

The data in S1 Table show the 141 individuals (85.5%) whose collected samples were sequenced. In the study, samples from 24 patients (14.5%) failed genotyping, among other causes due to non-amplification during RT-PCR and loss of cold chain during shipment to AIDS Research Laboratory (S1 Table, S1 Fig).

The median age was 31.6 years, with values ranging from 18 to 73 years. 80.9% of the patients belonged to the male sex and 76.3% were men who have sex with other men (MSM). The median viral load was 68,000 copies of RNA / mL and the CD4 + cell count was 371 cells / mm^3^ (Table 1). 14.9% of the patients studied had a CD4 + cell count below 200 cells / mm^3^. The predominant subtypes in the study population were subtypes B, CRF19_cpx and CRF20.23, 24_BG (S2 Fig).

**Table 1:** Demographic characteristics of 141 Cuban HIV-infected patients included in the study.

Characteristics	
**Age at sampling, years (IQR)**	31.6 (18–73)
**Age group, n (%)**	
≤25	41 (29.1)
>25	100 (70.9)
**Gender, n (%)**	
Men	114 (80.9)
Women	27 (19.1)
**Sexual orientation, n (%)**	
Men who have sex with other men (MSM)	87 (76.3)
Heterosexual (HT)	66 (34.9)
**HIV-1 viral load at sampling, RNA copies/ml, median, (IQR)**	68 000 (1090–1290000)
**CD4+ cell count at sampling, cells/mm**^**3**^**, median, (IQR)**	371 (9–1979)
**CD4+ cell count, n (%)**	
≤200	21 (14.9)
201–350	37 (26.2)
351–499	74 (52.5)
≥500	9 (6.4)
**Subtypes, n (%)**	
B	38 (26.9)
CRF19_cpx	34 (24.1)
CRF20, 23, 24_BG	33 (23.4)
CRF18_cpx	17 (12)
URF	13 (9.2)
C	3 (2.1)
A	1 (0.7)
G	2 (1.4)

The prevalence of PDR to any drugs classes was 29.8% (95% CI, 22.3–38.1). Drug resistance prevalence was 10.6% (95% CI 6.07–16.9) for any nucleoside reverse-transcriptase inhibitors (NRTIs), 23.4% (95% CI 16.7–31.3) for any non nucleoside reverse-transcriptase inhibitors (NNRTIs), 1.4% (95% CI 0.17–5.03) for any protease inhibitors (PIs). The most frequent mutations were K103N (12.1%); G190A (5.7%); Y181C (4.3%), which are associated with the high values of resistance to NNRTI described in the study (Table 2).

**Table 2:** Main mutations associated with HIV-1 resistance to antiretroviral drugs detected in Cuban patients during the national survey (January-June 2017).

Drug Resistance Mutations	Patients numbers	%
**NRTI**		
M41L	3	2.1
D67N	5	3.5
K70R	4	2.8
L74V	4	2.8
F77L	1	0.7
F116Y	1	0.7
M184V/I	4	2.8
L210W	3	2.1
T215Y	5	3.5
K219Q	6	4.3
**NNRTI**		
L100I	1	0.7
K101E	1	0.7
K103N	17	12.1
Y181C	6	4.3
G190A	8	5.7
M230L	4	2.8
**PI**		
D30N	1	0.7
M46I/L	2	1.4
V82A//T	1	0.7

The phylogenetic analysis of the 41 sequences with resistance associated mutations did not detect any transmission cluster (S3 Fig). In the phylogenetic tree as well as in the quality control of the sequences, three pairs of sequences with genetic distance lower than 0.5% were detected, which were: CUB-PDR-2017-CMG-0002 and CUB-PDR-2017-CMG -0003; CUB-PDR-2017-SCU-0015 and CUB-PDR-2017-SCU-0017; CUB-PDR-2017-SMP-0002 and CUB-PDR-2017-SMP-0003.

The prevalence of PDR was significantly higher in patients under 25 years of age (43.9%, 95% CI, 28.5–60.3, *p = 0*.*03*) compared to those over 25 years of age (24%, 95% CI, 16–33.6). Women presented a higher prevalence of PDR (28.9%, 95% CI, 22.3–61.2), followed by MSM (21.6%, 95% CI, 14.3–30.4) and heterosexual men (20.6%, 95% CI, 8.7–37.9). No association was found between the prevalence of PDR to the different families and combinations of ARVs and the different geographic regions of Cuba involved in the study (Table 3).

**Table 3:** Prevalence of pre-treatment resistance of HIV-1 in the geographic regions of Cuba.

Antiretrovirals	Geographic regions			
	Western * % (95%CI)	Havana ** % (95%CI)	Central *** % (95%CI)	Eastern **** % (95%CI)
NRTI	0	10.3 (4.6–19.4)	7.1 (0.2–33.9)	7.1 (0.2–19.5)
NNRTI	12.5 (0.3–52.6)	14.3 (7.3–24.1)	7.1 (0.2–33.9)	19 (8.6–34.1)
NRTI+NNRTI	0	5.2 (1.4–12.7)	0	2.3 (0.1–12.6)
NNRTI+PI	0	1.3 (0.0–7.0)	7.1 (0.2–33.9)	2.3 (0.1–12.6)
NRTI+NNRTI+PI	0	1.3 (0.0–7.0)	0	0

*-includes the Santa Cruz del Norte municipality belonging to the Mayabeque province

**-include the municipalities of the province of Havana (Plaza, San Miguel del Padrón, Habana Vieja, Cerro, Boyeros, Diez de Octubre, Arroyo Naranjo)

***- includes the Camagüey municipality of the Camagüey province

****- include the municipalities of Santiago de Cuba, Contramaestre and Palma Soriano of the Santiago de Cuba, the municipality of Manzanillo of Granma province, the municipality of Jobabo of Las Tunas province and the municipality of Guantánamo of Guantánamo province.

With respect to ARVs classes and therapeutic combinations, the detection of resistance to NNRTI in patients under 25 years of age was statistically significant (21.9%, 95% CI, 10.6–37.6, *p <0*.*0001*).CRF20, 23, 24_BG was the genetic variant with the highest prevalence of PDR (39.4%, 95% CI, 22.9–57.9), followed by CRF18_cpx (35.3%; 95% CI, 14.2–61.7), fundamentally to the NNRTI family.

**Fig 1:**
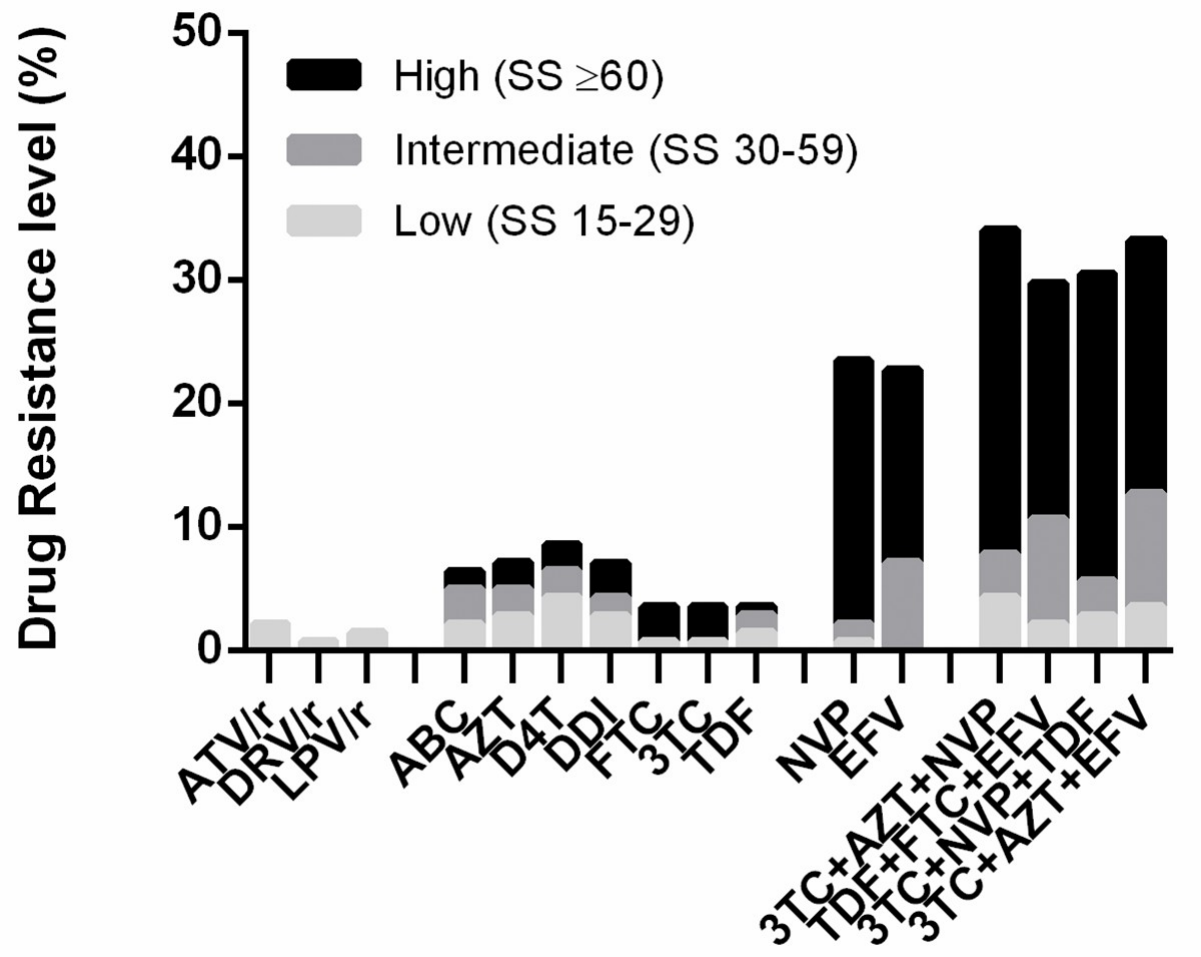
Levels of HIV-1 resistance to antiretrovirals. **Pre-treatment resistance levels of HIV-1 by antiretroviral drug were estimated from HIV-1 protease-RT sequences using the Stanford HIVdb tool algorithm (v 8.3). The levels of antiretroviral resistance according to the Stanford score (SS) were classified as: high, ≥60; intermediate, 30–50; or low (≥15–29). The pre-treatment resistance of HIV-1 is shown for the most frequently used treatment combinations.** ATV/r: atazanavir/ritonavir; DRV/r: darunavir/ritonavir; LPV/r: lopinavir/ritonavir; ABC: abacavir; AZT: zidovudine; D4T: stavudine; DDI: didanosine; FTC: emtricitabine; 3TC: lamivudine; TDF: tenofovir; NVP: nevirapine, EFV: efavirenz.

The determination of the levels of resistance to ARV combinations, according to the scoring values provided by the interpretation algorithm of the Stanford University Database (high and intermediate resistance), indicate that the treatment will not be effective in 29.7% of patients who start therapy with the lamivudine (3TC), zidovudine (AZT) and nevirapine (NVP) regimen. The therapeutic combination tenofovir (TDF), emtricitabine (FTC) and efavirenz (EFV), components of Atripla®, will not be effective in 27.6% of patients who initiate HAART (Fig 1).

## Discussion

The present work constituted the first study carried out in Cuba to know the prevalence of PDR of HIV-1 according to the methodology described by the WHO. The prevalence of PDR (29.8%) was higher than those described in previous studies conducted in Cuba during the 2007–2011 periods (12.5%) [9] and 2009–2012 (20.2%) [8]. Pre-treatment resistance prevalence values above 10% have been reported by countries that have implemented national surveys, such as Namibia, Nicaragua, Uganda, Zimbabwe and Argentina [13]. Other countries that implemented national surveys of pre-treatment HIV-1 resistance according to the WHO methodology showed a prevalence of less than 10%, such as Myanmar, Brazil, Mexico, Cameroon [13], South Africa [14] and Thailand [15]. Since 2001, Cuba has started HAART with the use of NRTI and NNRTI combinations produced by the national biopharmaceutical industry, which has benefited to 18744 PLHIV until December 2017. The sustained use of NVP or EFV in the first therapeutic line could favor the accumulation of mutations associated with resistance to these drugs (K103N, G190A and Y181C) and the subsequent transmission of the variants with these changes to the population. Several studies carried out in the pre-treatment population have described an increase in the prevalence of HIV-1 resistance to NNRTI since 2001, as the coverage of ARV therapy has increased. In studies conducted in several countries with the WHO methodology between 2014–2016, the prevalence of NNRTI in pre-treatment persons was higher in those who had previously been exposed to ARV (21.6%), compared with those not exposed (8.3%, p<0.0001) [13]. Non-adherence to HAART is a factor that contributes to the rapid emergence of resistant viruses, and the persistence of viral variants with mutations associated with resistance to ARVs in an individual, allows a prolonged "window of opportunity" for secondary transmission of resistant virus at the moment when the plasma viral load is high [16, 17]. The prevalence of pre-treatment resistance of HIV-1 in people less than 25 years of age in the present work indicates the transmission of resistant virus and its presence in the first years of infection [18]. However, variants with mutations associated to resistance in chronically infected individuals were detected, which supports what has been referred by several authors about the persistence of resistant virus from three to ten years in untreated individuals [16, 19]. In addition, the presence of resistant virus in sanctuary sites and the ability to integrate with the DNA of the host cell favor the persistence of HIV-1 variants resistant to ARVs [20].14.9% of the patients had CD4 + cell count values below 200 cells / mm^3^, indicating the marked depression of the immune system. In this group of patients, the possible re-infection with genetic variants of HIV-1 different from the one causing the primary infection and presenting mutations associated to resistance to ARVs in their genome may be one of the causes of existing of pre-treatment resistance. Several studies have associated a higher prevalence of HIV-1 resistance mutations in subtype B than in non-B subtypes [21, 22]. In the present study, association between the viral variants described and the presence of resistance mutations was not found; however, a greater presence of mutations was detected in the CRF20, 23, 24_BG, which has increased its circulation in the Cuban seropositive population [7, 9, 23]. In the study, transmission cluster was not detected; however, three pairs of sequences had genetic distances less than 0.5. When analyzing the epidemiological history of the patients to whom the sequences corresponded, a close epidemiological relationship was found, since they were in contact with one another, which evidences the transmission of the same viral variant of HIV.

One of the limitations presented by the study was the failure of the genotyping of a group of samples from the municipalities of the eastern region, Contramaestre and Palma Soriano, belonging to the Santiago de Cuba province. The loss of the cold chain during the process of transportation to AIDS Research Laboratory could be one of the causes of the decrease in plasma viral load at values below the limit of sensitivity of the genotypic assays for the determination of HIV drug resistance. The use of dried blood spots (DBS) would be a useful sample to avoid the loss of the cold chain during transportation to the genotyping laboratory. However, this type of sample was not used in the national survey, because at the time of the study, the test for the determination of HIV drug resistance from DBS was not validated in the AIDS Research Laboratory. The results of the national survey show that the combinations of drugs used in the first line of treatment will not be effective in 29.7% that start with 3TC + AZT + NVP and 27.6% of those that start with Atripla, so it was valued the change of treatment strategies in Cuba. WHO recommends not using NNRTIs in the first line of therapy if the PDR to this family of drugs is greater than 10% and, if this is not possible, consider introducing pre-treatment HIVDR testing [24]. The non-prescription of NNRTI and the incorporation of dolutegravir (DTG), an integrase inhibitor, into the first-line therapeutic combinations in Cuba since the fourth quarter of 2018, was one of the quick responses of the Ministry of Public Health to face the HIVDR [25]. Likewise, WHO has described in the Global Action Plan on HIV Drug Resistance, 2017–2021, a group of interventions to be developed, and investment of resources at a global and country level to guide the response to this problem [24]. The systematic monitoring of early warning indicators of HIVDR and educational and prophylactic interventions regarding the good adherence to treatment in PLHIV, mainly among young people and women, will allow maintaining the achievement achieved by Cuba to be the first country in the world to eliminate the transmission of HIV from mother to child and contribute to meet the third 90 of goals 90, 90, 90, enunciated by the WHO for the year 2020, which is to ensure that 90% or more of PLHIV with HAART reach undetectable HIV-1 viral load.

## Conclusions

In the present study, an increase in pre-treatment resistance of HIV-1 to NNRTIs in Cuban patients who participated in the national survey was described, which evidenced the need to assess the change to more optimal and effective therapeutic strategies and strengthen the actions of prevention and surveillance of HIVDR.

## Supporting information

S1 Table Distribution of samples of pre-treatment HIV-1 Cuban patients among the municipalities of Cuba according PPPS sampling. (DOCX)

S1 Fig Geographical map of Cuba indicating the 15 municipalities that contributed samples to the national survey. (TIF)

S2 Fig Phylogenetic tree of the HIV-1 pol gene sequences of the 141 samples analyzed in the present study. The samples are indicated by the symbol ● and the reference sequences of the subtypes and CRF were obtained from the Los Alamos database. The tree was constructed by the maximum likelihood method and the genetic distance was estimated according to the Kimura 2 parameter model. Numbers near the nodes represent bootstrap values (1,000 replicas).
(TIF)

S3 Fig Phylogenetic tree including all 41 sequences with at least one SDRM. No clustering is observed among these samples. The letters PDR-2017 indicate the Cuban sequences.
(TIF)
