# Supplementary material for: National survey of pre-treatment HIV drug resistance in Cuban patients
Source: PLoS One. 2019 Sep 3;14(9):e0221879. doi: 10.1371/journal.pone.0221879 (PMC6719847; doi:10.1371/journal.pone.0221879)
Supplement: S1 Table — (DOCX) [file pone.0221879.s001.docx]

**S1 Table**. **Distribution of samples of pre-treatment HIV-1 Cuban patients among the municipalities of Cuba according PPPS sampling**

| **Municipalities** | **Samples numbers** | **Samples numbers received** | **Samples number sequenced** | **%** | **Samples numbers Unsuccessfully Sequenced** | **%** |
| --- | --- | --- | --- | --- | --- | --- |
| Santiago de Cuba | 18 | 18 | 13 | 72.2 | 5 | 27.8 |
| Camagüey | 14 | 14 | 14 | 100 | 0 | 0 |
| 10 de Octubre | 11 | 11 | 11 | 100 | 0 | 0 |
| San Miguel del Padrón | 11 | 11 | 11 | 100 | 0 | 0 |
| Guantánamo | 11 | 11 | 11 | 100 | 0 | 0 |
| Boyeros | 11 | 11 | 11 | 100 | 0 | 0 |
| Plaza | 11 | 11 | 11 | 100 | 0 | 0 |
| Arroyo Naranjo | 11 | 11 | 11 | 100 | 0 | 0 |
| Habana Vieja | 11 | 11 | 11 | 100 | 0 | 0 |
| Cerro | 11 | 11 | 11 | 100 | 0 | 0 |
| Palma Soriano | 11 | 11 | 4 | 36.4 | 7 | 63.6 |
| Contramaestre | 11 | 11 | 2 | 18.2 | 9 | 81.8 |
| Manzanillo | 11 | 11 | 8 | 72.7 | 3 | 27.3 |
| Santa Cruz del Norte | 8 | 8 | 8 | 100 | 0 | 0 |
| Jobabo | 4 | 4 | 4 | 100 | 0 | 0 |
| **Total** | 165 | 165 | **141** | 85.5 | 24 | 14.5 |
