# Supplementary figures and images for: National survey of pre-treatment HIV drug resistance in Cuban patients
Source: PLoS One. 2019 Sep 3;14(9):e0221879. doi: 10.1371/journal.pone.0221879 (PMC6719847; doi:10.1371/journal.pone.0221879)

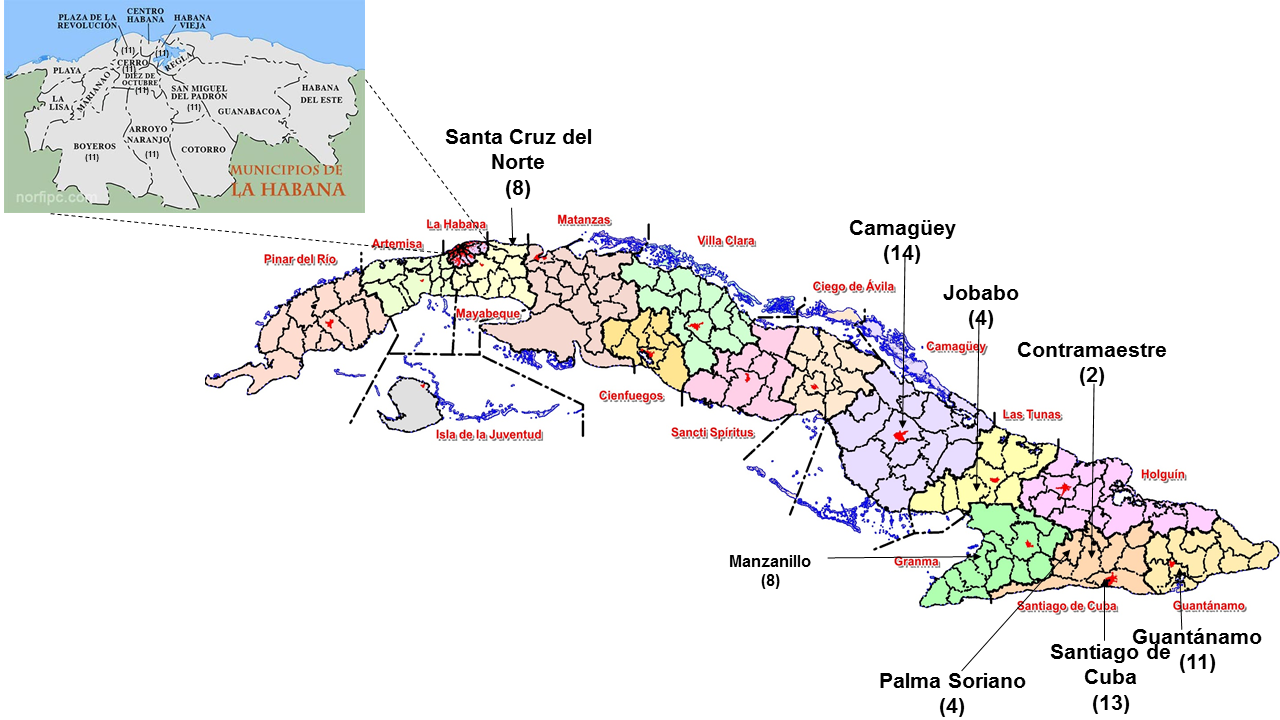

Supplement: S1 Fig — (TIF) [file pone.0221879.s002.tif]

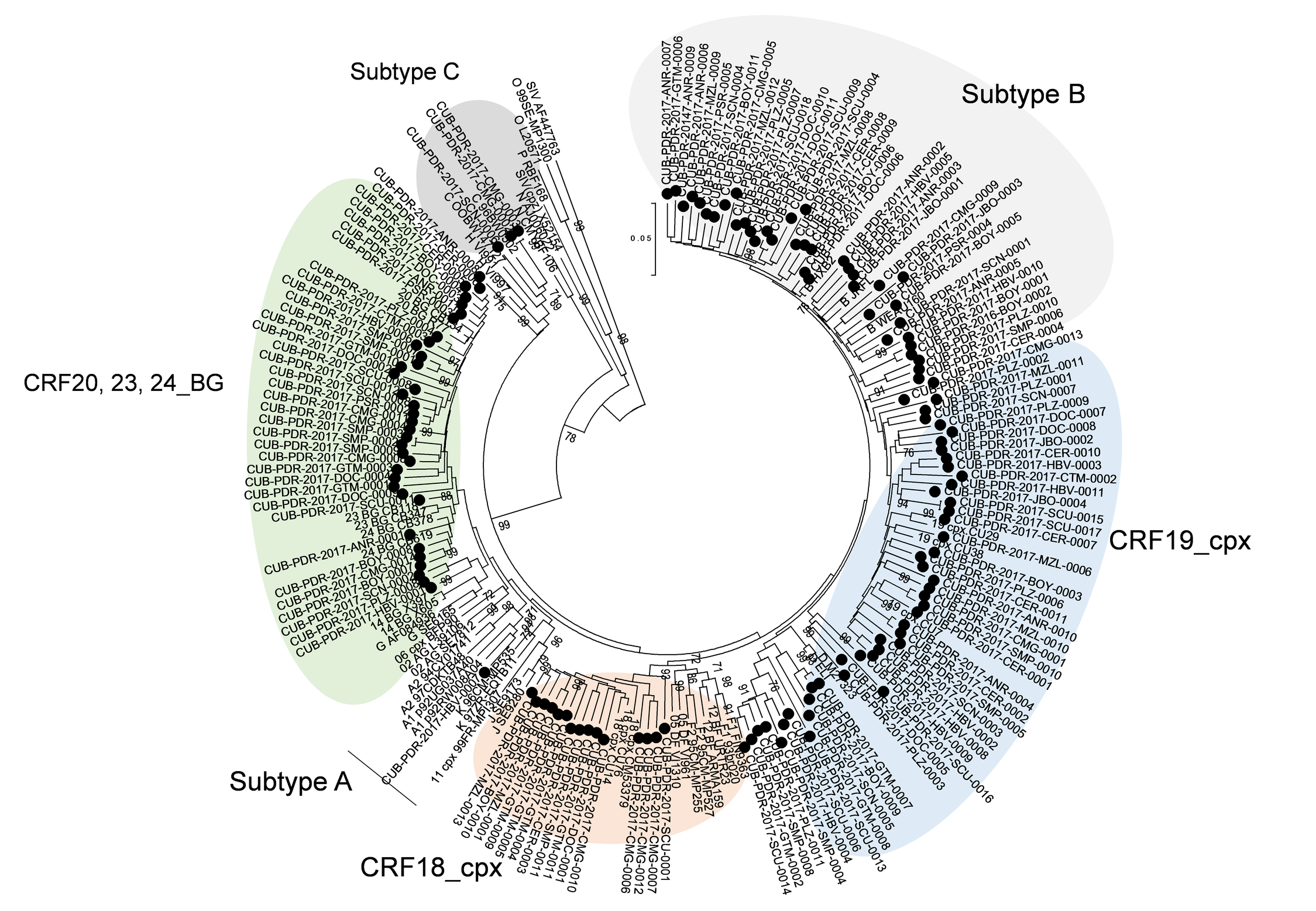

Supplement: S2 Fig — The samples are indicated by the symbol ● and the reference sequences of the subtypes and CRF were obtained from the Los Alamos database. The tree was constructed by the maximum likelihood method and the genetic distance was estimated according to the Kimura 2 parameter model. Numbers near the nodes represent bootstrap values (1,000 replicas). (TIF) [file pone.0221879.s003.tif]

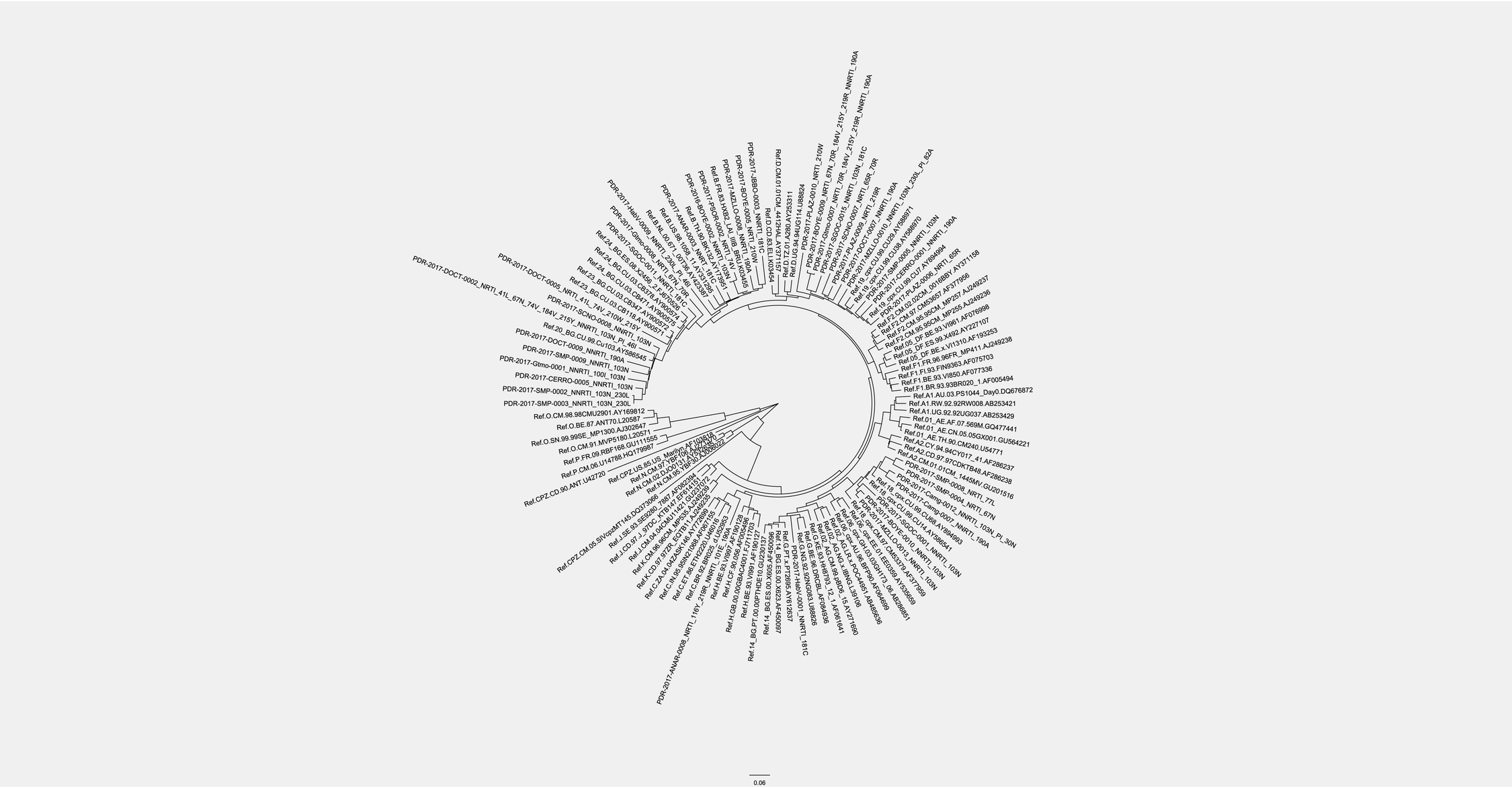

Supplement: S3 Fig — No clustering is observed among these samples. The letters PDR-2017 indicate the Cuban sequences. (TIF) [file pone.0221879.s004.tif]
